# Association between Urban Educational Policies and Migrant Children’s Social Integration in China: Mediated by Psychological Capital

**DOI:** 10.3390/ijerph20043047

**Published:** 2023-02-09

**Authors:** Cixian Lv, Peijin Yang, Jingjing Xu, Jia Sun, Yuelong Ming, Xiaotong Zhi, Xinghua Wang

**Affiliations:** Normal College, Qingdao University, Qingdao 266071, China

**Keywords:** educational policies, migrant children, psychological capital, social integration

## Abstract

China’s urban educational policies have been established to solve the problems of potential discrimination and inequitable access to education, disrupting migrant children who move from rural areas to urban cities and who tend to suffer from a range of mental health issues. However, little is known regarding how China’s urban educational policies affect migrant children’s psychological capital and social integration. This paper aims to explore the effect of urban education policies on improving migrant children’s psychological capital level in China. The second objective of this paper is to examine whether policies can encourage them to integrate into urban society in a positive way. This paper thoroughly analyzes the impact of China’s urban educational policies on three dimensions of social integration of migrant children (identification, acculturation, and psychological integration), and also verifies the mediating effects of psychological capital on the relationships between these variables. The subjects of this study are 1770 migrant children in grades 8–12, who are sourced from seven coastal cities in China. Multiple regression analysis and mediation effect tests are employed to analyze the data. This study reveals that migrant children’s identification with educational policies has a significant positive impact on their psychological capital. Psychological capital has partial mediating effects on the relationship between identification with educational policies and the three dimensions of social integration. In other words, identification with educational policies indirectly affects the process of social integration of migrant children through psychological capital. Based on this, for the purpose of promoting the positive impacts of educational policies of inflow cities on the social integration of migrant children, this study makes the following recommendations: (a) at the micro-level, the psychological capital of individual migrant children should be enhanced; (b) at the meso-level, the partnerships between migrant children and urban children should be taken seriously; and (c) at the macro-level, the urban educational policies related to migrant children should be improved. This paper not only makes policy recommendations for improving the educational policies of inflow cities, but also offers a Chinese perspective on the research related to the tricky issue facing all countries around the world, the social integration of migrant children.

## 1. Introduction

In the context of China’s reform and opening up, and the rural revitalization strategy, China is experiencing a process of rapid urbanization and the large-scale migration of the population. According to the results of China’s Seventh National Population Census, China’s total population in 2020 was 1412 million, including 376 million migrants. The number of migrant children in China was 71.09 million, including 14.6225 million interprovincial migrant children [1]. China’s Ministry of Education defines “migrant children” as school-age children who live with their migrant worker parents in inflow cities and receive compulsory education there, but their household registrations are still in other provinces or other cities in the same province [1]. In China, migrant education is limited by the household registration system. In other words, a student should attend the primary school and high school in the place where his/her household is registered. With the relaxation of the policy, migrant children are allowed to attend some public school in inflow cities. However, the number and quality of schools need to be improved. At the same time, there are different cultural characteristics between the urban society where migrant children move to and the rural society where they are born, such as different cultural customs, lifestyles, school environments, and courses offered. Thus, in addition to geographical migration, similar to the children of international immigrants, migrant children in China also face a culture shock. It is challenging for them to adapt to urban society, so they become “marginal persons” in urban society.

Robert Park, an American scholar, proposed an acculturation model for immigrant children. He believes that the individual experiencing acculturation always lies at a certain point in the transition from the complete original culture to the complete mainstream culture, and will eventually be assimilated by the mainstream culture [2]. Based on the attitudes of the individual experiencing acculturation towards the original culture and the mainstream culture, John Berry proposed two separate dimensions, namely “willingness to preserve the original culture and identity” and “willingness to communicate with different cultural groups”. In other words, an individual’s high identification with a certain culture does not mean he/she will have low identification with other cultures. In addition, John Berry also proposed four different acculturation strategies: integration, assimilation, separation, and marginalization [3]. Many international studies have revealed that immigrant children in inflow cities lack social support and suffer from multiple forms of discrimination and exclusion, which adversely affects their mental health and thus hampers the social integration process in inflow cities [4,5]. When inflow countries cannot provide effective integration education for immigrant children, it becomes difficult for them to integrate into urban society. At the same time, they struggle with relationships, mental health, and self-identity as they integrate at school [6]. Zlobina et al. (2006) surveyed 518 first-generation immigrants living in Spain and found that perceived social discrimination demonstrated a significant positive prediction effect on the acculturation dilemma of immigrant children. In order to obtain higher quality education and more room for future development, China’s migrant children move with their migrant worker parents from remote rural villages in central and western China to coastal cities in eastern China, to live and study there [7]. However, they suffer from discrimination and exclusion from urban society, and inequitable access to education, etc., which has led to the development of a range of mental health issues [8,9]. As such, the research on migrant children’s educational attainment and social integration is of great practical significance.

In light of the analysis above, what does the psychological capital and social integration of migrant children in China look like after moving with their migrant worker parents from rural areas to cities? Have urban educational policies that act as social support contributed to raising the level of migrant children’s psychological capital? Do these policies encourage migrant children to integrate into urban society in a positive way? As China’s educational policies and measurement index systems continue to be improved, can they provide reference significance for research on the social integration and educational attainment of immigrant children in other countries? Improved public educational policies are expected to change the attitudes of mainstream society members towards migrant children. This study provides policy support for Chinese migrant children’s educational attainment and social integration in inflow cities.

This study used the stratified sampling method to select samples from coastal cities in eastern China where migrant children are relatively concentrated. We collected 1770 valid questionnaires. Through the joint use of multiple regression analysis and mediating effect tests, the present study analyzes the positive effects of educational policies on the social integration of migrant children, as well as the mediating effects of psychological capital on the relationships between these variables.

## 2. Theoretical Framework and Hypotheses

### 2.1. The Connotation of Social Integration and Its Influencing Factors

Henri Tajfel (1986) proposed the social identity theory from the perspective of macro-social integration and social psychology. In his opinion, “social identity” refers to when an individual becomes aware of a specific social group or social category he/she belongs to. At the same time, the individual also realizes the emotional and value significance provided to him/her by being a member of the group [10]. Ellemers et al. (1988) believe that social identity theory can explain the background, intrinsic motivation, strategy selection, and other issues, in the emergence of group behaviors [11]. Canino et al. (2022) argue that social cognitive theory can explain the subjective differences in how individuals feel [12]. Existing studies on the social integration of international immigrants and their children are mainly carried out in such subjects as sociology, psychology, and pedagogy. These studies provide references for us to explore the theoretical construction, connotation dimensions, measurement methods, and factors, influencing the social integration of migrant children in China. Moen and Williams (1989) argue that social integration is the cohesion of social groups, including social psychological (or emotional) integration and structural integration [13]. Bosswick and Heckmannh (2006) defines “social integration” as a dynamic process of interactions between immigrants and inflow cities [14]. Wang et al. (2022) believes that the process of social integration not only reflects the physical and mental health, and development of migrant children, but also reflects the level of urban social integration and stable development [15]. This study argues that the social integration of migrant children in China is a process of gradual internalization. Migrant children first generate identification with and emotional belonging to the values, ways of life, ways of behavior, and ways of thinking of urban society. Finally, such identification and emotional belonging gradually leads to internalization by those individuals.

Based on analysis of the existing literature, academic circles believe that the factors affecting the social integration of migrant children mainly include the macro-social structure, the educational policies, the characteristics of the school where the child is enrolled, the family background of the migrant children, and the personality traits of the migrant children. Firstly, the education systems and supporting policies of inflow cities in China are some of the most important factors affecting the social integration of migrant children [16]. Secondly, the educational philosophy, the teacher–student relationship, and the socializing among peers of different social classes in the schools of inflow cities have significant effects [17,18]. Thirdly, the social capital, the educational expectations, and the parent–child relationships of migrant children families are also key influencing factors [19,20]. Finally, many of the psychological characteristics of individual migrant children, such as their mood, personality, self-concept, self-esteem, mental control, and happiness, directly or indirectly affect the state of their social integration [21,22].

### 2.2. The Relationship between Social Integration and Migrant Children’s Identification with Educational Policies

More and more researchers believe that the degree of social integration of migrants is influenced by the social structure and social policies of inflow cities. According to the social identity theory, there are two types of subjective belief systems in society, namely social change, and social mobility [10]. The belief in social change is based on a rigid social structure. In other words, the boundaries between groups are stable and difficult to penetrate. An individual cannot leave his/her original group and enter a superior group. The belief in social mobility is based on a resilient and permeable social structure. In other words, an individual has the opportunity to move from one group to another. Weber (1978) argues that there are various closed mechanisms of social mobility in modern industrial society, and such mechanisms impede intergenerational and intragenerational mobility of social classes [23]. As a result, the superior classes are able to maximize their interests. The Chinese government has always placed a great deal of value on the education of migrant children, and has introduced a series of urban educational policies. Unlike general educational policies, the characteristics of urban educational policies are as follows: (1) the government departments of inflow cities are mainly responsible for solving the urban schooling problem of migrant children; (2) the full-time public primary and secondary schools in inflow cities are mainly responsible for receiving migrant children; and (3) the residence permit of migrant children, rather than their household registration, is used as the main certificate for enrollment [1]. The studies of Xu et al. (2022) argue that migrant children’s perceptions of fairness and equity from urban educational policies help them enjoy high-quality urban education, realize higher educational attainment and accumulation of human capital, and ultimately promotes their social integration [24]. On the basis of the analysis above, this study develops the following hypotheses on the relationship between social integration and migrant children’s identification with educational policies:

**Hypothesis** **1a** **(H1a).** *Migrant children’s identification with educational policies has a significant impact on their identification with social integration*;

**Hypothesis** **1b** **(H1b).** *Migrant children’s identification with educational policies has a significant impact on their acculturation for social integration*;

**Hypothesis** **1c** **(H1c).** *Migrant children’s identification with educational policies has a significant impact on their psychological integration for social integration*.

### 2.3. The Relationship between Psychological Capital and Migrant Children’s Identification with Educational Policies

The ecological systems theory argues that individual development is embedded in a series of environmental systems that influence each other [25]. In other words, the interaction between individuals and environmental systems, and the interaction among environmental systems, jointly affect the development of individual cognition, behavior, and other aspects [25]. According to this theory, the environmental subsystems, from the inside to the outside, are the microsystem, the mesosystem, the exosystem, and the macrosystem [26]. Meanwhile, many studies have shown that, as a potential positive psychological force for an individual, psychological capital is impacted by many factors, which not only include the individual’s own physical and psychological characteristics, but also include peer groups, families, schools, and macro-social environments [27,28]. This paper is mainly based on the impact of macro-educational policies on the psychological capital of the sample. Demerath et al. (2008) analyzed the data from the survey on American suburban high school students and concluded that a positive community environment has a positive catalytic effect on the development of students’ psychological capital; conversely, it is not conducive to the healthy development of students’ psychological capital [29]. Huang and Zhang (2021) argue that there is a certain intrinsic relationship between a sense of social support and psychological capital, that is, the sense of social support can positively affect students’ psychological capital, which is a positive condition for improving the level of psychological capital [30]. The educational policies of inflow cities have gradually eliminated migrant children’s sense of discrimination and loneliness, and have reinforced their hopes and beliefs on integration into urban society. As a result, the implementation of policies creates an opportunity for migrant children to leapfrog from a vulnerable group to a dominant group. According to the aforementioned information, this study advances the following hypothesis on the relationship between psychological capital and migrant children’s identification with educational policies:

**Hypothesis** **2** **(H2).** *Migrant children’s identification with educational policies has a significant impact on their psychological capital*.

### 2.4. The Relationship between Migrant Children’s Psychological Capital and Social Integration

In recent years, studies on psychological capital have gradually expanded to the field of youngsters. Zhang et al. (2010) reveal that psychological capital is significantly correlated with the test subjects’ self-esteem, internal control, emotional balance, and other indicators reflecting their level of mental health [31]. Gu et al. (2019) have shown that psychological capital is conducive to the social integration and career success of new immigrants in urban areas, and thus promotes the sustainable development of society [32]. Among the studies on the social integration of migrant children, people are gradually realizing that there is a greater need to develop the positive psychological capital of the relocated children of migrant workers, so that they can overcome adversity. Wang et al. (2014) surveyed migrant children aged 7–16 and found that: (1) the migrant children attending public schools scored higher on the psychological quality dimension than those attending migrant schools; (2) the interpretation range of positive psychological quality compared to the variation rate of scores of various dimensions of environmental adaptation is from 17% to 26% [33]. In summary, psychological capital and its elements have a wide range of positive effects on the psychological and behavioral adaptation of the populations concerned. Although there are few people involved in current studies on the correlation between migrant children’s psychological capital and social integration (including several dimensions of social integration), we can draw inspiration from the above studies.

However, according to a review of the relevant literature and actual surveys, it can be found that the introduction and implementation of educational policies do not have the same impact on the social integration of all migrant children. Does this mean that there may be other influencing factors in the relationship between educational policies and social integration? Through a survey of 187 male youth volunteers, Boen and Vanbeselae (2000) argue that whether a group is willing to try to leapfrog from a vulnerable group to a dominant group is influenced by the interaction of boundary permeability and individual characteristics [34]. When the group boundary is fully or partially open, only those individuals who feel they are capable enough are willing to try to leapfrog to the dominant group. Through a questionnaire survey of 1350 migrant children currently attending the seventh grade in China, Lv et al. (2022) show that differences in identification have partial mediating effects between urban educational policies and psychological integration, and cultural integration [16]. It can be seen from this, that the urban education policies of inflow cities (the independent variables) have a direct impact on the social integration of migrant children (the dependent variable), and are also indirectly influenced by psychological capital (the mediating variable).

According to the analyses above, the present study develops the following hypotheses on the relationship between migrant children’s identification with educational policies and psychological capital, and social integration:

**Hypothesis** **3a** **(H3a).** *Migrant children’s psychological capital has a significant positive impact on their identification*;

**Hypothesis** **3b** **(H3b).** *Migrant children’s psychological capital has a significant positive impact on their acculturation*;

**Hypothesis** **3c** **(H3c).** *Migrant children’s psychological capital has a significant positive impact on their psychological integration*;

**Hypothesis** **4a** **(H4a).** *Migrant children’s psychological capital has a mediating effect between their identification and their identification with educational policies*;

**Hypothesis** **4b** **(H4b).** *Migrant children’s psychological capital has a mediating effect between their identification with educational policies and their acculturation*;

**Hypothesis** **4c** **(H4c).** *Migrant children’s psychological capital has a mediating effect between their identification with educational policies and their psychological integration*.

**Figure 1:**
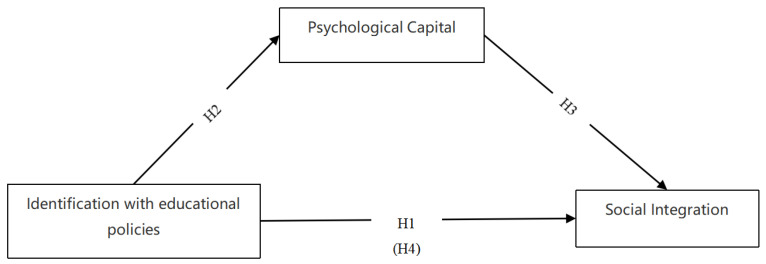
Hypothesized model.

Based on the above research hypotheses, this study develops a theoretical model (see Figure 1).

## 3. Research Design and Measurement of Variables

### 3.1. Research Samples and Data Collection

This study employed the stratified sampling method. The target samples were recruited from seven cities with a high number of migrant children in China, including Dalian City in Liaoning Province, Qingdao City in Shandong Province, Nanjing City in Jiangsu Province, Hangzhou City in Zhejiang Province, Xiamen City in Fujian Province, Guangzhou City in Guangdong Province, and Haikou City in Hainan Province. The samples of migrant children were the students in grades 8–12 in 12 schools in these seven cities. All of them were the direct targets of policy implementation. With the use of on-site screening of questionnaires and the listwise deletion approach, the research team obtained a total of 1770 valid questionnaires, with a recovery rate of 82.3%. Table 1 below presents the descriptive statistics of the questionnaire.

Among the samples surveyed, the average age was 16 years old; 45.9% of the samples were female, while 54.1% of the samples were male; 79.4% of the migrant children were moved from a village to a city, while 20.6% of the migrant children were moved from a city to another city. The fathers of 95.6% of the migrant children received a senior high school education or below, while the fathers of 4.4% of the migrant children received an undergraduate education or above. In terms of the type of school attended, 75.1% of the migrant children attended local public schools, while 24.9% of the migrant children attended designated migrant schools. In addition, 80.1% of the migrant children had lived in inflow cities for more than three years, and 61.9% of the migrant children had lived in inflow cities for more than five years.

### 3.2. Variable Definition and Measurement

This paper designed a five-point Likert scale, in which “1” represented “Strongly Disagree” and “5” indicated “Strongly Agree” [35]. There were three variables, including the “Scale of Psychological Capital” [36], the “Scale of Migrant Children’s Social Integration” [37,38], and the “Scale of Identification with Educational Policies” [39].

#### 3.2.1. Migrant Children’s Psychological Capital (PC)

Psychological capital is a positive state of mind that individuals gradually develop and exhibit as they grow [31]. In this study, “migrant children’s psychological capital” refers to the characteristics children show in such dimensions as self-confidence, optimism, hope, and resilience in their school studies and family life. Currently, there is no research and development on the scale of migrant children’s psychological capital. This study draws on the international youth psychological capital scale and measures migrant children’s psychological capital in four dimensions, including self-confidence, optimism, resilience and hope [36]. On the basis of the original scale, this scale has added or adjusted the items that can reflect the characteristics of Chinese migrant children. There are 16 items in the scale of Chinese migrant children’s psychological capital. For example, “I am very confident in my current learning ability”. The Cronbach’s alpha for the scale of psychological capital is 0.945. Among others, there are four items on self-confidence, for which the Cronbach’s alpha is 0.913; there are four items on optimism, for which the Cronbach’s alpha is 0.893; there are four items on resilience, for which the Cronbach’s alpha is 0.916; and there are four items on hope, for which the Cronbach’s alpha value is 0.892. The CITC value for each measurement item is higher than 0.5. A confirmatory factor model of migrant children’s psychological capital has been conducted. The composite reliabilities of the four dimensions are 0.951, 0.925, 0.908 and 0.917, respectively, while the construct validities are 0.711, 0.673, 0.682 and 0.576, respectively. The two-order four-factor confirmatory factor model basically fits the research data.

#### 3.2.2. Migrant Children’s Social Integration (SI)

“Integration” refers to the process by which migrants gradually adapt to the mainstream system of inflow cities in such dimensions as economy, behavior, culture, and concept [14]. In fact, social integration implies an unequal principal–subordinate relationship in terms of culture and behavior, that is, the culture of the inflow place is in a dominant position, while the culture of the immigrant is in a vulnerable position [3]. The measurement index system of migrant children’s social integration mainly includes the following dimensions: (1) surface integration, where the individuals gradually accept the language, food, and clothing of the people in inflow cities; (2) meso-level integration, where the individuals gradually converge with the customs, behaviors and thinking of the people in inflow cities; and (3) deep integration, where the individuals have developed a deep understanding of the culture, ideology, and values of the people in inflow cities [40]. Some studies argue that social integration mainly includes four dimensions: psychological integration, cultural integration, social interaction integration, and identity integration [41]. This study argues that Chinese migrant children’s social integration is in essence a social action, and a complex process of active adaptation, rather than a one-way process of passive acceptance. Different from that of adults, migrant children’s social integration has unique characteristics. In particular, indicators such as occupation and income cannot be observation dimensions of migrant children’s social integration. Therefore, on the basis of previous studies, this study mainly selects three core measurement dimensions: identification, acculturation, and psychological integration. Through a review of the literature, the scale of migrant children’s identification for social integration mainly draws on the acculturation model index of Ward and Rana-Deuba [37]. On this basis, items in the scale have been added or adjusted to reflect the characteristics of Chinese migrant children. The scale of Chinese migrant children’s identification for social integration consists of nine items, mainly involving their preference for the city in which they live, their willingness to obtain urban household registration, and their self-identification with the urban identity. For example, “I feel that I am already an urban person”. The scale of migrant children’s acculturation for social integration draws on the socio-cultural adaptation scale of Ward and Kennedy [42], based on which items in the scale have been added or adjusted to reflect the characteristics of Chinese migrant children. The scale of Chinese migrant children’s acculturation for social integration consists of nine items, mainly involving the degree of dialect mastery, customs familiarity, and the participation of migrant children in community cultural activities. For example, “I can accept local etiquette or rituals”. The scale of migrant children’s psychological integration for social integration draws on the internationally accepted Self-Esteem Scale (SES) [38]. Based on this scale, items in the scale have been added or adjusted to reflect the characteristics of Chinese migrant children. The scale of Chinese migrant children’s psychological integration for social integration consists of nine items, mainly involving the loneliness and anxiety, self-values, and peer–to–peer compatibility of migrant children. For example, “I often feel lonely and helpless in the city”. In this study, the Cronbach’s alpha for the scale of migrant children’s identification for social integration is 0.910; the Cronbach’s alpha for the scale of migrant children’s acculturation for social integration is 0.956; and the Cronbach’s alpha for the scale of migrant children’s psychological integration for social integration is 0.926. Each measurement item’s CITC value is higher than 0.5. We have constructed a confirmatory factor model of migrant children’s social integration. The combined reliabilities of the dimensions are 0.913, 0.933, and 0.912, respectively, while the construct validities are 0.751, 0.632, and 0.583, respectively. The first-order three-factor confirmatory factor model satisfies the research data.

#### 3.2.3. Migrant Children’s Identification with Educational Policies (IEP)

The educational policies for migrant children in inflow cities are mandatory macro-social policies, the impact of which on the targets of policy implementation varies to a certain extent. Parsons’ systems theory provides the method for measurement and dimension division of the identification with policies [39]. Migrant children’s identification with educational policies, a variable, is essentially the cognitive level, the degree of recognition, and the value judgment of migrant children about the relevant educational policies of inflow cities [16,24]. Based on the above literature, starting from three dimensions (policy awareness, policy recognition, and policy evaluation), this study has prepared the scale of Chinese migrant children’s identification with educational policies. Through small-sample predictions, and data reliability and validity tests, the items were refined. For example, “I think the educational policies of inflow cities are fair and equitable”. In the end, the scale of Chinese migrant children’s identification with educational policies includes a total of nine items. The Cronbach’s alpha for the scale of Chinese migrant children’s identification with educational policies is 0.955. Each measurement item’s CITC value is higher than 0.5. We have constructed a confirmatory factor model of migrant children’s identification with educational policies, the combined reliability of which is 0.873, while the construct validity is 0.536. The first-order confirmatory factor model satisfies the research data.

### 3.3. Data Analysis

Using correlation analysis, the present study initially validated the correlation between migrant children’s identification with educational policies, and psychological capital, and social integration. Then, this paper tested the mediating effects through regression analysis, focusing on the mediating effects of psychological capital between identification with educational policies (the variable) and social integration (the variable). Since migrant children’s social integration differs by varying degrees in relation to demography, we included demographics as a control variable in the regression model. To summarize, through using the aforementioned methods, this study analyzes the positive effects of migrant children’s identification with educational policies on social integration.

## 4. Results

### 4.1. Correlation Analysis

The correlation analysis outcomes of migrant children’s identification with educational policies, psychological capital, and social integration are shown in Table 2. Among others, there is a significant positive correlation between migrant children’s identification with educational policies (IEP) and the four dimensions of psychological capital (PC), as follows: self-confidence (*r* = 0.686 **, *p* < 0.01), optimism (*r* = 0.849 **, *p* < 0.01), resilience (*r* = 0.784 **, *p* < 0.01), and hope (*r* = 0.767 **, *p* < 0.01). There is a significant positive correlation between migrant children’s identification with educational policies (IEP) and the dimensions of social integration (SI), specifically as follows: identification (*r* = 0.896 **, *p* < 0.01), acculturation (*r* = 0.874 **, *p* < 0.01), and psychological integration (*r* = 0.600 **, *p* < 0.01). There is a significant positive correlation between the dimension of self-confidence in psychological capital (PC) and the different dimensions of social integration (SI): identification (*r* = 0.664 **, *p* < 0.01), acculturation (*r* = 0.649 **, *p* < 0.01), and psychological integration (*r* = 0.599 **, *p* < 0.01).

### 4.2. Regression Analysis

In order to ensure that the multi-linear regression model can come up with a scientific and reasonable explanation, we have to examine the multicollinearity, serial correlation, heteroscedasticity of the model, etc. The multicollinearity test was conducted using the tolerance and variance inflation factor (VIF). In the present study, each variable’s VIF in each model ranges from 0 to 3, showing a minimal multicollinearity problem. Since the present study does not entail the comparison of multi-period sample values, and the Durbin–Watson (DW) value of each regression model is approximately 2, there is little chance to commit the serial correlation issue. The scatter plot of the residual term is used to determine the heteroskedasticity problem. In the present study, the scatter plot of each regression model is in a disordered state, and the heteroscedasticity problem usually appears in time-series data, so we judge that there is no heteroscedasticity problem in this study.

#### 4.2.1. Analysis of the Regression of Migrant Children’s Identification with Educational Policies on Their Social Integration

In the regression model, the dependent variables include migrant children’s identification, acculturation, and psychological integration for social integration. The control variables include gender, the type of household registration flow, the father’s level of education, the father’s occupation type, and the time of inflow into the city. The independent variable is the migrant children’s identification with educational policies. The results reveal that migrant children’s identification with educational policies has a significant positive impact on their identification for social integration (*β* = 0.864, *p* < 0.01), thus supporting H1a. Migrant children’s identification with educational policies has a significant positive impact on their acculturation for social integration (*β* = 0.799, *p* < 0.01), therefore substantiating H1b. Migrant children’s identification with educational policies has a significant positive impact on their psychological integration for social integration (*β* = 0.742, *p* < 0.01). As such, H1c is supported by the data. As a result, migrant children’s identification with educational policies has a significant positive impact on their social integration.

#### 4.2.2. Analysis of the Regression of Migrant Children’s Identification with Educational Policies on Their Psychological Capital

In the regression model, the dependent variables include the self-confidence, optimism, resilience, and hope of migrant children’s psychological capital. The control variables include gender, the type of household registration flow, the father’s level of education, the father’s occupation type, and the time of inflow into the city. The independent variable is the migrant children’s identification with educational policies. The results reveal that migrant children’s identification with educational policies has a significant positive impact on the level of self-confidence in their psychological capital (*β* = 0.721, *p* < 0.01). Migrant children’s identification with educational policies has a significant positive impact on the level of optimism in their psychological capital (*β* = 0.881, *p* < 0.01), the level of resilience in their psychological capital (*β* = 0.832, *p* < 0.01), and the level of hope in their psychological capital (*β* = 0.802, *p* < 0.01). Therefore, migrant children’s identification with educational policies demonstrates a significant positive influence on their psychological capital. Hypothesis H2 is supported by the data.

#### 4.2.3. Testing of the Mediating Effects of Psychological Capital between Identification with Educational Policies and Social Integration

In this study, the procedures for testing the mediating effects of psychological capital between identification with educational policies and social integration are as follows. The first step is to test *Y* = c*X* + e_1_, whether c is significant. The second step is to test *M* = a*X* + e_2_ and *Y* = c′*X* + b*M* + e_3_, whether a and b are significant. If both a and b are significant, the third step test shall be conducted to determine whether c′ (partial mediating effect or complete mediating effect) is significant; if neither a nor b is significant, the test shall be stopped; and if only a or b is significant, the Sobel test shall be performed. From the previous regression analysis, it can be seen that the regression coefficient of migrant children’s identification with educational policies on their social integration is positive and significant, and the regression coefficient of migrant children’s identification with educational policies on their psychological capital is positive and significant. Therefore, c in the equation *Y* = c*X* + e_1_ is significant, and a in the equation *M* = a*X* + e_2_ is significant.

##### Testing of the Mediating Effects of Psychological Capital between Identification and Identification with Educational Policies

Table 3 shows the results of testing the mediating effects of psychological capital between identification and identification with educational policies. The explanatory powers of eight hierarchical models are: 0.596, 0.821, 0.697, 0.820, 0.661, 0.824, 0.615 and 0.819, respectively. All variables can explain 81.9% of the variance of the dependent variable. It is found via the F test that the explanatory powers are statistically significant.

In Table 3, migrant children’s identification for social integration is a dependent variable. Model 1 shows that self-confidence, as a variable comprising psychological capital, has a significant positive prediction on identification (*β* = 0.689, *p* < 0.01). Model 3 shows that optimism has a significant positive prediction on identification (*β* = 0.735, *p* < 0.01). Model 5 shows that resilience has a significant positive prediction on identification (*β* = 0.689, *p* < 0.01). Model 7 shows that the variable of hope has a significant positive prediction for identification (*β* = 0.663, *p* < 0.01). As a result, all of the four dimensions of migrant children’s psychological capital have a significant positive prediction on their identification for social integration (the dependent variable). The research hypothesis H3a is verified.

In Table 3, Model 2, Model 4, Model 6, and Model 8 are employed to test whether b and c′ in the equation *Y* = c′*X* + b*M* + e_3_ are significant. Model 2 shows that the regression coefficient of the variable of self-confidence comprising migrant children’s psychological capital on their identification is still positively significant (*β* = 0.121, *p* < 0.01). Model 4 shows that the regression coefficient of the optimism variable of psychological capital on identification is still positively significant (*β* = 0.144, *p* < 0.01). Model 6 shows that the regression coefficient of the resilience variable of psychological capital on identification is still positively significant (*β* = 0.158, *p* < 0.01). Model 8 shows that the regression coefficient of the hope variable of psychological capital on identification is still positively significant (*β* = 0.102, *p* < 0.01). Thus, this study shows that b in the equation *Y* = c′*X* + b*M* + e_3_ is significant. Meanwhile, Model 2 shows that the regression coefficient for migrant children’s identification with educational policies on their identification is positively significant (*β* = 0.777, *p* < 0.01). Model 4 shows that the regression coefficient of identification with educational policies on identification is positively significant (*β* = 0.737, *p* < 0.01). Model 6 shows that the regression coefficient of identification with educational policies on identification is positively significant (*β* = 0.732, *p* < 0.01). Model 8 shows that the regression coefficient of identification with educational policies on identification is positively significant (*β* = 0.782, *p* < 0.01). Thus, this study shows that c′ in the equation *Y* = c′*X* + b*M* + e_3_ is significant. It can be seen from this, that psychological capital has mediating effects between identification and identification with educational policies. Therefore, the research hypothesis H4a is supported by the data.

##### Testing of the Mediating Effects of Psychological Capital between Acculturation and Identification with Educational Policies

Table 4 presents the outcomes of testing the mediating effects of psychological capital between acculturation and identification with educational policies. The explanatory powers of eight hierarchical models are: 0.608, 0.789, 0.680, 0.794, 0.592, 0.791, 0.791, 0.563, and 0.791, respectively. All variables can explain 79.1% of the variance of the dependent variable. It is found by the F test that the explanatory powers are statistically significant.

In Table 4, migrant children’s acculturation for social integration is a dependent variable. Model 1 shows that the self-confidence variable of psychological capital has a significant positive prediction on acculturation (*β* = 0.654, *p* < 0.01). Model 3 shows that the variable of optimism has a significant positive prediction on acculturation (*β* = 0.683, *p* < 0.01). Model 5 shows that the variable of resilience has a significant positive prediction on acculturation (*β* = 0.597, *p* < 0.01). Model 7 shows that the variable of hope has a significant positive prediction on acculturation (*β* = 0.572, *p* < 0.01). As a result, all of the four dimensions of migrant children’s psychological capital have a significant positive prediction on their acculturation for social integration (the dependent variable). The research hypothesis H3b is verified.

In Table 4, Model 2, Model 4, Model 6, and Model 8 are employed to test whether b and c′ in the equation *Y* = c′*X* + b*M* + e_3_ are significant. Model 2 shows that the regression coefficient of the variable of self-confidence to acculturation is still positively significant (*β* = 0.131, *p* < 0.01). Model 4 shows that the regression coefficient of the variable of optimism to acculturation is still positively significant (*β* = 0.113, *p* < 0.01). Model 6 shows that the regression coefficient of the variable of resilience to acculturation is still positively significant (*β* = 0.121, *p* < 0.01). Model 8 shows that the regression coefficient of the variable of hope to acculturation is still positively significant (*β* = 0.102, *p* < 0.05). Thus, this study shows that b in the equation *Y* = c′*X* + b*M* + e_3_ is significant. Meanwhile, Model 2 shows that the regression coefficient for children’s identification with educational policies to acculturation is positively significant (*β* = 0.709, *p* < 0.01). Model 4 shows that the regression coefficient of children’s identification with educational policies to acculturation is positively significant (*β* = 0.699, *p* < 0.01). Model 6 shows that the regression coefficient of children’s identification with educational policies to acculturation is positively significant (*β* = 0.698, *p* < 0.01). Model 8 shows that the regression coefficient of children’s identification with educational policies to acculturation is positively significant (*β* = 0.715, *p* < 0.01). Thus, this study shows that c′ in the equation *Y* = c′*X* + b*M* + e_3_ is significant. It can be seen from this, that the four dimensions of migrant children’s psychological capital have mediating effects between their identification with educational policies and their acculturation for social integration. Therefore, the research hypothesis H4b is supported by the data.

##### Testing of the Mediating Effects of Psychological Capital between Psychological Integration and Identification with Educational Policies

Table 5 presents the results of testing the mediating effects of psychological capital between psychological integration and identification with educational policies. The explanatory powers of eight hierarchical models are: 0.634, 0.724, 0.699, 0.731, 0.674, 0.730, 0.650 and 0.725, respectively. All variables can explain 72.5% of the variance of the dependent variable. The F test shows that the explanatory powers are statistically significant.

In Table 5, migrant children’s psychological integration for social integration is a dependent variable. Model 1 shows that the self-confidence variable of psychological capital has a significant positive prediction on psychological integration (*β* = 0.642, *p* < 0.01). Model 3 shows that the optimism variable of psychological capital has a significant positive prediction on psychological integration (*β* = 0.685, *p* < 0.01). Model 5 shows that the resilience variable of psychological capital has a significant positive prediction on psychological integration (*β* = 0.647, *p* < 0.01). Model 7 shows that the hope variable of psychological capital has a significant positive prediction on psychological integration (*β* = 0.622, *p* < 0.01). As a result, all of the four dimensions of migrant children’s psychological capital have a significant positive prediction on their psychological integration for social integration (the dependent variable). The research hypothesis H3c is verified.

In Table 5, Model 2, Model 4, Model 6, and Model 8 are employed to test whether b and c′ in the equation *Y* = c′*X* + b*M* + e_3_ are significant. Model 2 shows that the regression coefficient of the variable of self-confidence to psychological integration is still positively significant (*β* = 0.221, *p* < 0.05). Model 4 shows that the regression coefficient of the variable of optimism to psychological integration is still positively significant (*β* = 0.327, *p* < 0.01). Model 6 shows that the regression coefficient of the variable of resilience to psychological integration is still positively significant (*β* = 0.273, *p* < 0.01). Model 8 shows that the regression coefficient of the variable of hope to psychological integration is still positively significant (*β* = 0.224, *p* < 0.01). Thus, this study shows that b in the equation *Y* = c′*X* + b*M* + e_3_ is significant. Meanwhile, Model 2 shows that the regression coefficient for migrant children’s identification with educational policies to psychological integration is positively significant (*β* = 0.583, *p* < 0.01). Model 4 shows that the regression coefficient of migrant children’s identification with educational policies to psychological integration is positively significant (*β* = 0.454, *p* < 0.01). Model 6 shows that the regression coefficient of migrant children’s identification with educational policies to psychological integration is positively significant (*β* = 0.515, *p* < 0.01). Model 8 shows that the regression coefficient of migrant children’s identification with educational policies to psychological integration is positively significant (*β* = 0.552, *p* < 0.01). Thus, this study shows that c′ in the equation *Y* = c′*X* + b*M* + e_3_ is significant. It can be seen from this, that the four dimensions of migrant children’s psychological capital have mediating effects between their identification with educational policies and their psychological integration for social integration. Therefore, the research hypothesis H4c is supported by the data.

## 5. Discussion

International researchers have focused on the correlation among the urban environment, psychological capital, and social integration of immigrant children in destination countries. Studies argue that immigrant children’s psychological capital is generally not high, and their social integration is generally not good. Therefore, scholars believe that research needs to further explore the relationship between the urban environment, psychological capital, and social integration [5,34,36]. This study has also found that the overall level of psychological capital and social integration of Chinese migrant children in inflow cities is not high. The underlying reason is that most migrant children are suffering from implicit discrimination in the educational policies, social welfare, cultural customs, and other aspects of inflow cities, leaving them in a state of being “marginal persons” in cities. John Berry classified acculturation strategies using individual immigrant’s attitudes towards their original groups and current new groups during the acculturation process, and proposed four acculturation strategies, including integration, assimilation, separation, and marginalization [3].

(1) This study finds that although migrant children have lived in inflow cities, they still try to maintain a clear “safe distance” from the local mainstream culture. The strategy of acculturation marginalization of migrant children refers to when they dissociate from both urban and rural cultures. In these instances, neither do they persistently identify with the original rural culture, nor do they actively integrate into the mainstream culture of inflow cities. According to social identity theory, the higher the service quality provided by the urban government, the more it can promote the social integration of migrant children [43]. This study has further found that a series of educational policies of inflow cities have had significant positive impacts on such dimensions as migrant children’s identification, acculturation, and psychological integration for social integration (see Table 2 of this study). Regression analysis reveals that migrant children’s identification with educational policies also has significant positive impacts on the social integration dimensions, such as their identification, acculturation, and psychological integration. These research conclusions support the mainstream view of international research, that is, the more institutional support the society provides, the higher the overall degree of social integration of immigrants.

(2) Through regression analysis, this study has found that migrant children’s identification with educational policies demonstrates a significant positive influence on their psychological capital. To be more specific, migrant children’s recognition, perception of fairness, and perception of influence on the educational policies of inflow cities, have significant impacts on such psychological capital dimensions as self-confidence, optimism, resilience, and hope. However, different from the previous research conclusions [29,44], the mediating effect test in this study shows that in addition to the direct effect, identification with educational policies also has an indirect effect through the mediating variable “psychological capital” (see Table 3, Table 4 and Table 5).

Therefore, the importance of this study is reflected in the fact that both the research data and the hypothesis tests prove that identification with urban educational policies contributes to improving the level of migrant children’s psychological capital (including self-confidence, hope, optimism, and resilience), thus promoting their social integration in inflow cities.

### 5.1. Implications

For the purpose of providing full play to the positive impacts of China’s urban educational policies and the psychological capital of individual migrant children on their social integration, this study makes the following recommendations:

Firstly, at the micro-level, we should enhance the psychological capital of individual migrant children. Migrant children themselves need to try their best to overcome such negative attitudes as cowardice and inferiority, actively demonstrate their strengths, and actively integrate into school and society. Migrant children should have clear learning goals and strive to improve their self-learning ability. In addition, migrant children should also take the initiative to seek help from teachers and classmates and strive to improve their learning ability. Migrant children’s good academic performance can effectively enhance their psychological capital. Parents of migrant children should be patient and tolerant in dealing with their children’s integration problems with school studies and urban life. They should imperceptibly influence their children’s social integration through their own behaviors. They should establish good parent–child relationships and listen more to their children’s confusion and joy in studying and living in the city. Schools should pay attention to the growth process of individual migrant children and help them build their self-confidence and a sense of accomplishment.

Secondly, at the meso-level, we should attach importance to establishing good partnerships between migrant children and urban children. According to the ecological systems theory, the peer–to–peer relationship is important in the process of individual development. Previous studies have found that if migrant children often communicate with their peers who are keen on learning, their enthusiasm for educational expectations, learning input, and social integration, will also be significantly improved [45,46]. Moreover, a good peer–to–peer relationship can promote the social integration of migrant children through interpersonal communication, mental health, academic achievements, and other aspects [47,48]. Therefore, the schools in inflow cities need to further break the segregation between the students of different social classes. Schools should encourage migrant children to actively participate in school learning activities and to get to know various types of partners. In addition, schools should be committed to creating a harmonious campus environment and class atmosphere for children of different social classes, for the purpose of fostering good peer–to–peer relationships among migrant children. Good peer–to–peer relationships can help migrant children better integrate into urban society.

Thirdly, at the macro-level, the urban management authorities in inflow cities should further revise and improve the relevant educational policies. Firstly, urban management authorities should adhere to the governance philosophy of safeguarding social fairness and justice, and break the “ceiling effect” of the household registration system, for the purpose of gradually realizing the equal treatment for urban and rural residents. Secondly, urban management authorities should further improve corresponding supporting policies relating to migrants’ healthcare, employment, community services, and their children’s schooling, etc. Thirdly, urban management authorities should respond positively to migrant children’s education demands. Fair and just educational policies in inflow cities can greatly promote the learning enthusiasm and social integration of migrant children. As a result, migrant children would benefit from their educational attainment and accumulation of human capital. This is of great significance to the sustainable development of China’s economy and society in the future.

### 5.2. Contributions

The first contribution of this study is that it offers a Chinese perspective on the research on this issue and enriches the research content in this area. In the eastern coastal region of China, the educational policies of big inflow cities have significantly affected the social integration of migrants (especially migrant children). This study considers that Chinese migrant children’s psychological capital may have an impact on their social integration process. Therefore, this paper incorporates the variables of psychological capital (including resilience, optimism, self-confidence, and hope) into the regression model.

The second contribution of this study is that it has developed a multidimensional approach to measuring the degree of social integration of migrant children. Based on the social identity theory, the states of migrant children’s identification and acculturation are the key indicators to measure their social integration. However, migrant children in grades 8–12 are in a psychologically sensitive period and are vulnerable to factors such as the social environment, the perception of discrimination, and the sense of deprivation. Therefore, this study focused on analyzing the psychological characteristics and psychological integration of migrant children in China.

The third contribution of this study is that it is of great practical significance. At the micro-level, the social integration status of migrant children is closely related to their individual growth and development. At the macro-level, the mental health and personal development of migrant children have always been the issues that require long-term attention and deep concern in the field of education in China. Urban educational policies aim to provide equitable educational opportunities for migrant children. The present study not only provides new evaluation support for the implementation of educational policies, but also offers theoretical support and policy recommendations for practical issues, such as the social integration of migrant children in China.

### 5.3. Limitations and Future Research

The present study has the following limitations. Firstly, the sample size of this study is limited. The selection of the research sample is based on the principles of accessibility and convenience, resulting in the issue of generalizability of the research results. Future research may consider using large-scale Chinese data survey platforms such as the China Education Panel Survey (CEPS) and Chinese Family Panel Studies (CFPS) to embed some scale on the identification with China’s educational policies and other aspects for larger scale research. Secondly, the experimental hypotheses are insufficient. This paper focuses on the differential analysis of control variables, such as the type of household registration flow, the father’s education level, and the father’s occupation type, but it has not taken into account the heterogeneity of migrant children of different ages, nationalities, and social classes. Future studies are advised to further improve the experimental design and consider more potential influencing factors, and continuously make still further progress on the experimental design.

## 6. Conclusions

Migrant children in China move with their migrant worker parents from rural areas to cities. This paper aims to explore the effect of urban education policies on improving migrant children’s psychological capital level in China. The second objective of this paper is to examine whether policies can encourage them to integrate into urban society in a positive way. Under the urban–rural dual structure, although migrant children have lived in inflow cities, they still try to maintain a clear “safe distance” from the local mainstream culture. This study has further found that a series of educational policies of inflow cities have had significant positive impacts on such dimensions as migrant children’s identification, acculturation, and psychological integration for social integration. Using regression analysis, this study has found that migrant children’s identification with educational policies has a significant positive impact on their psychological capital. Further regression analysis reveals that migrant children’s identification with educational policies also has significant positive impacts on the social integration dimensions, such as their identification, acculturation, and psychological integration. The mediating effect test of this study shows that in addition to the direct effect, identification with educational policies also has an indirect effect through the mediating variable “psychological capital”. This study can be useful for the improvement of urban educational policies related to migrant children and to maintain the psychological wellbeing of migrant children who move from rural areas to urban cities and are struggling to adjust to the new environments.

## Figures and Tables

**Table 1:** Demographic information of the sample.

Variables	*N* (%)	Distribution of Samples						
		ZJ	FJ	JS	SD	LN	HN	GD
Sex								
Female	813 (45.9)							
Male	957 (54.1)							
Type of household mobility								
“Rural-urban” mobility	1405 (79.4)	278	225	186	156	174	183	203
“Town-town” mobility	365 (20.6)	75	80	66	35	39	38	32
School type								
Voluntary school (migrant children)	441 (24.9)	65	30	55	39	36	58	59
Local public school	1329 (75.1)	218	135	138	147	101	113	102
Father’s level of education								
High school level or below	1625 (95.6)	203	277	247	209	184	266	239
Undergraduate degree or above	75 (4.4)	11	13	8	12	17	9	5
Duration of residence								
1–3 years	352 (19.9)	74	52	33	27	42	64	76
3–5 years	322 (18.2)	75	67	60	45	39	15	21
≥5 years	1096 (61.9)	183	128	141	159	224	115	146

Note. *N* = 1770; ZJ = Zhe Jiang; FJ = Fu Jian; JS = Jiang Su; SD = Shan Dong; LN = Liao Ning; HN = Hai Nan; GD = Guang Dong.

**Table 2:** Correlation analysis of values.

	(1)	(2)	(3)	(4)	(5)	(6)	(7)	(8)
(1) IEP	1							
(2) Self-Confidence	0.686 **	1						
(3) Optimistic	0.849 **	0.755 *	1					
(4) Resilience	0.784 **	0.737 **	0.813 **	1				
(5) Hope	0.767 **	0.755 **	0.793 **	0.827 **	1			
(6) Identification	0.896 **	0.664 **	0.796 **	0.749 **	0.724 **	1		
(7) Acculturation	0.874 **	0.649 **	0.764 **	0.669 **	0.654 **	0.834 **	1	
(8) PI	0.600 **	0.599 **	0.644 **	0.637 **	0.599 **	0.504 **	0.416 **	1

Note. *N* = 1770; IEP = identification with educational policies; PI = psychological integration; * *p* < 0.05, ** *p* < 0.01.

**Table 3:** Moderating effect of PC on the relationship between IEP and Identification.

Variables	Identification							
	Model 1	Model 2	Model 3	Model 4	Model 5	Model 6	Model 7	Model 8
Sex	0.021	0.028	0.019	0.023	0.004	0.029	0.052	0.022
Type of household mobility	0.085 *	0.043	0.095 *	0.049	0.124 *	0.054	0.115 *	0.048
Type of School	−0.030	−0.048	0.051	0.054	0.057	0.052	0.062	0.055 *
Father’s Level of Education	0.065	0.043	0.089 *	0.050	0.123 *	0.057 *	0.131 *	0.054
Duration of Residence	0.208 *	0.028	0.062	0.004	0.044	0.002	0.050	0.001
IEP		0.777 **		0.737 **		0.732 **		0.782 **
Self-Confidence	0.689 **	0.121 **						
Optimistic			0.735 **	0.144 **				
Resilience					0.689 **	0.158 **		
Hope							0.663 **	0.102 **
Δ*R*^2^	0.596	0.821	0.697	0.820	0.661	0.824	0.615	0.819
F	137.654 **	200.114 **	194.068 **	207.231 **	169.194 **	206.830 **	149.408 **	200.960 **

Note. IEP = identification with educational policies; PC = psychological capital; * *p* < 0.05, ** *p* < 0.01.

**Table 4:** Moderating effect of PC on the relationship between IEP and Acculturation.

Variables	Acculturation							
	Model 1	Model 2	Model 3	Model 4	Model 5	Model 6	Model 7	Model 8
Sex	0.081 *	0.036	0.081	0.040	0.076	0.039	0.116 *	0.038
Type of household mobility	0.088 *	0.049	0.099 *	0.055 *	0.130	0.052	0.121 *	0.051
School type	−0.033	−0.016	−0.010	−0.007	−0.004 *	−0.001	−0.008	−0.001
Father’s Level of Education	0.077	0.057	0.101 *	0.063	0.132 *	0.059	0.139 *	0.058 *
Duration of Residence	0.129 *	0.037 *	0.011	0.067 *	0.031	0.077	0.026	0.030
IEP		0.709 **		0.699 **		0.698 **		0.715 **
Self-Confidence	0.654 **	0.131 **						
Optimistic			0.683 **	0.113*				
Resilience					0.597 **	0.121 *		
Hope							0.572 **	0.105 *
Δ*R*^2^	0.608	0.789	0.680	0.794	0.592	0.791	0.563	0.791
F	338.447 **	713.918 **	451.717 **	699.618 **	307.280 **	684.365 **	277.910 **	684.796 **

Note. IEP = identification with educational policies; PC = psychological capital; * *p* < 0.05, ** *p* < 0.01.

**Table 5:** Moderating effect of PC on the relationship between IEP and PI.

Variables	PI							
	Model 1	Model 2	Model 3	Model 4	Model 5	Model 6	Model 7	Model 8
Sex	0.078	0.042	−0.076	0.051	0.063	0.040	0.107 *	0.055
Type of household mobility	0.102 *	0.134 *	0.093 *	−0.121 *	0.066	0.114 *	0.075	0.122 *
Type of School	−0.013	−0.001	−0.007	−0.008	−0.012	0.009	−0.016	−0.011
Father’s Level of Education	0.085	0.101 *	0.062	−0.086	0.031	−0.077	−0.023	0.078
Duration of Residence	0.073	0.060 *	0.063	0.098 *	0.080	0.109 *	0.074	0.110 *
IEP		0.583 **		0.454 **		0.515 **		0.552 **
Self-Confidence	0.642 **	0.221 *						
Optimistic			0.685 **	0.327 **				
Resilience					0.647 **	0.273 **		
Hope							0.622 **	0.224 **
Δ*R*^2^	0.634	0.724	0.699	0.731	0.674	0.730	0.650	0.725
F	311.001 **	825.824 **	473.668 **	824.866 **	400.963 **	845.162 **	332.368 **	818.248 **

Note. IEP = identification with educational policies; PI = psychological integration; PC = psychological capital; * *p* < 0.05, ** *p* < 0.01.

## Data Availability

The data presented in this study are available on request from the corresponding author. The data are not publicly available due to ethical reasons.
